# Targeting brain-peripheral immune responses for secondary brain injury after ischemic and hemorrhagic stroke

**DOI:** 10.1186/s12974-024-03101-y

**Published:** 2024-04-18

**Authors:** Mingxu Duan, Ya Xu, Yuanshu Li, Hua Feng, Yujie Chen

**Affiliations:** 1 Department of Neurosurgery, State Key Laboratory of Trauma, Burn and Combined Injury, Southwest Hospital, Third Military Medical University (Army Medical University), 29 Gaotanyan Street, Shapingba District, Chongqing, 400038 China; 2 Chongqing Key Laboratory of Intelligent Diagnosis, Treatment and Rehabilitation of Central Nervous System Injuries, Southwest Hospital, Third Military Medical University (Army Medical University), Chongqing, 400038 China; 3 Chongqing Clinical Research Center for Neurosurgery, Southwest Hospital, Third Military Medical University (Army Medical University), Chongqing, 400038 China

**Keywords:** Brain–peripheral crosstalk, Stroke, Inflammatory infiltration, Meningeal lymphatic vessel, Choroid plexus

## Abstract

The notion that the central nervous system is an immunologically immune-exempt organ has changed over the past two decades, with increasing evidence of strong links and interactions between the central nervous system and the peripheral immune system, both in the healthy state and after ischemic and hemorrhagic stroke. Although primary injury after stroke is certainly important, the limited therapeutic efficacy, poor neurological prognosis and high mortality have led researchers to realize that secondary injury and damage may also play important roles in influencing long-term neurological prognosis and mortality and that the neuroinflammatory process in secondary injury is one of the most important influences on disease progression. Here, we summarize the interactions of the central nervous system with the peripheral immune system after ischemic and hemorrhagic stroke, in particular, how the central nervous system activates and recruits peripheral immune components, and we review recent advances in corresponding therapeutic approaches and clinical studies, emphasizing the importance of the role of the peripheral immune system in ischemic and hemorrhagic stroke.

## Introduction

Because of the tolerance of the central nervous system (CNS) to antigen-induced immune responses, coupled with the presence of specific physical barriers, such as the blood‒brain barrier (BBB) and the blood‒cerebrospinal fluid barrier (BCSFB), which isolate the CNS from the peripheral immune system, the CNS has always been regarded as an immune-privileged system, and the presence of a peripheral immune component in the CNS in a healthy state has been considered a manifestation of pathology. However, over the past 20 years, this perception has changed more objectively [1]. Increasing evidence suggests that the CNS and the peripheral immune system are not two completely isolated systems but rather represent a complex network of interactions [2, 3]. Indeed, the peripheral immune system maintains surveillance of the CNS, and it not only recognizes external pathogens but also plays an important role in aseptic CNS injury, such as ischemic/hemorrhagic stroke and traumatic brain injury (TBI) [4–6]. In the past, researchers have focused on exploring the pathophysiological mechanisms underlying these primary sterile CNS injury lesions, such as the sudden cessation of blood supply to vascular regions in ischemic stroke leading to the death of nerve cells to form ischemic cores, the sudden overflow of vascular blood components in cerebral hemorrhage, and, in more complex subarachnoid hemorrhage (SAH), hypoxia following hemorrhage [7, 8]. However, while targeted treatment partially controls further injury, the poor long-term prognosis and lower survival rates have led a growing number of investigators realizing that secondary injury following primary injury may play an important role in long-term prognosis [9].

Thus, the role of the peripheral immune system in secondary damage following CNS injury has important implications for the long-term prognosis of the injury. Indeed, a more accurate description of the whole process is that the CNS already activates and recruits components of the peripheral immune system after the onset of injury through a variety of pathways, including the autonomic nervous system, the neuroendocrine system, and the meningeal lymphatics; these components enter the CNS through a variety of modalities, such as the BBB and the BCSFB; and these components exert both deleterious and beneficial effects through interactions with the intrinsic cells of the CNS. Therefore, targeted monitoring and intervention of the immune system not only are important for the prediction of disease progression but also hold promise for improving long-term neurological prognosis and reducing mortality.

## Brain–peripheral crosstalk in inflammatory infiltration after stroke

As a central regulator of organisms, the brain is responsible for maintaining the homeostasis of organisms, while the advantages and significance of CNS regulation of the peripheral immune system are mainly reflected in the following aspects. First, the brain constantly monitors the internal and external environment and integrates this information to regulate the immune system in synchronization with the cardiovascular system and digestive system to play a beneficial role under physiological and pathological conditions. Second, an important role of the brain is to predict threats and coordinate the body’s response after damage occurs, which requires the brain to play a strong regulatory role in numerous systems that protect and repair the body, including the immune system. Finally, a significant advantage of the nervous system over the immune system is its speed of response, even within milliseconds of injury. In response to injury, the CNS responds quickly and powerfully to modulate immune responses in an effective manner [2]. We next explored the interaction between the CNS and the peripheral immune system after injury.

### Peripheral immune responses induced by stroke (Fig. 1)

**Fig. 1:**
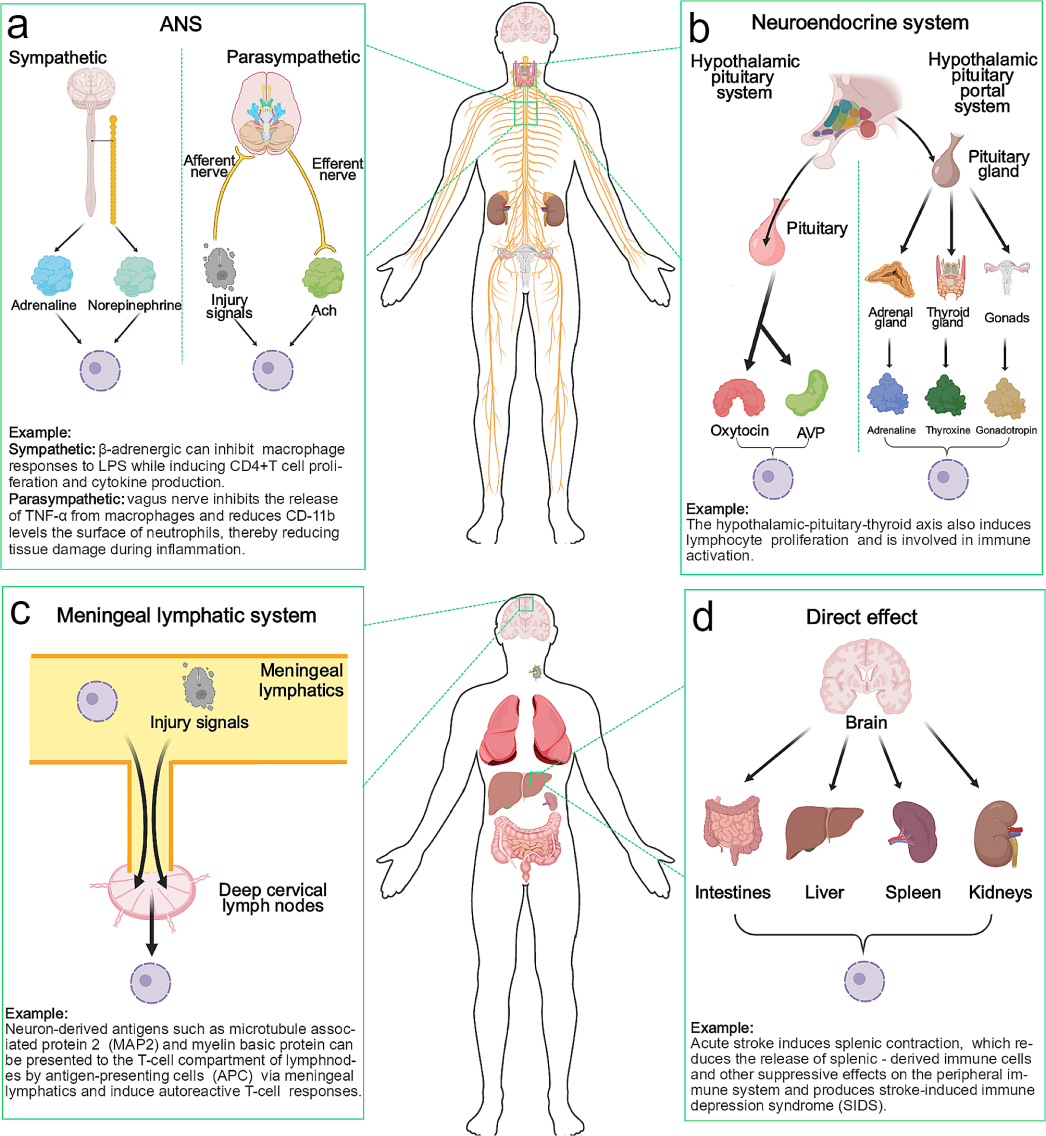
Schematic diagram of peripheral immune responses induced by hemorrhagic stroke

#### Autonomic nervous system (ANS)

The most direct influence is that of the neural signaling output network, in which the sympathetic nervous system (SNS) network and the parasympathetic nervous system (PSNS) network play major roles [10]. The most obvious role of sympathetic nerves is to help the body produce a stress response, and these nerves are the main source of epinephrine and norepinephrine after CNS injury. Most importantly, immune cells express both α-adrenergic and β-adrenergic receptors, and the level of receptor expression correlates with cellular status and whether the cells are mature or activated [11, 12]. Through the activation of these receptors, the CNS can influence immune cell migration and cytokine production [13–15], but these effects are likely to be double-edged swords because recent research has shown contradictions in terms of whether this effect is beneficial [16]. For example, β-adrenergic receptor activation can play a role in inducing CD4^+^ T-cell proliferation and cytokine production [17, 18] while inhibiting macrophage responses to lipopolysaccharide (LPS) [19]. In addition, it has been demonstrated that the norepinephrine produced by sympathetic nerves in the spleen can partially activate memory T cells and that the acetylcholine produced by activated T cells can in turn inhibit cytokine production in macrophages [3].

An important component of the parasympathetic nervous system is the vagus nerve. The afferent arc of the vagus nerve senses the stimulation of inflammatory cells and secretes ACH in the efferent arc in the periphery, thereby suppressing the inflammatory response [20]. In autoimmune diseases, it has also been shown that suppressing the immune response by stimulating the vagus nerve can be therapeutic in rheumatoid arthritis [21]. Specifically, macrophages are the first peripheral immune cells shown to be affected by the vagus nerve, which inhibits the release of TNF-α from macrophages [22–24]. In addition, the vagus nerve reduces the level of CD11b on the surface of neutrophils, thus attenuating tissue damage during inflammation [25]. However, the interactions between the nervous system and the immune system are extremely complex. The most direct evidence is that immune cells themselves can secrete different neuronal factors [26] and that many functions of the vagus nerve are mediated through sympathetic nerves [27]. To further complicate matters, sympathetic influences on peripheral inflammation can even differ on different sides of the body [28], and in arthritic patients with stroke, the antigen-specific response of T cells is significantly stronger in the limb on the side affected by the stroke [29], suggesting that there may be differences in the regulation of the peripheral immune system in each of the different cerebral hemispheres [30–33].

#### Neuroendocrine system

Cutting-edge research has confirmed that the neuroendocrine system is also an important way in which the nervous system achieves central-to-peripheral regulation. The neuroendocrine system consists of the hypothalamic neuropituitary system, which primarily secretes oxytocin and arginine vasopressin (AVP), and the hypothalamic-pituitary portal system, which secretes adrenocorticotropic hormone (ACTH), thyroid stimulating hormone (TSH), follicle-stimulating hormone (FSH), luteinizing hormone (LH), and growth hormone (GH) in an endocrine manner [34–36]. Oxytocin and AVP in the hypothalamic-pituitary system have been shown to correlate with immunoreactivity after injury; specifically, oxytocin inhibits proinflammatory cytokines [37, 38], and the anti-inflammatory effects of AVP are mediated through the activation of AVP-producing neurons by inflammatory factors [39, 40]. In the hypothalamic-pituitary portal system, sex steroids are involved in the programming of the immune system [41], and testosterone is immunosuppressive, whereas estrogen is, in contrast, immunopotentiating [42]. The hypothalamic-pituitary-thyroid axis has also been shown to induce lymphocyte proliferation and to be involved in immune activation [43, 44], which explains the diminished immune response after thyroidectomy [45]. However, the specific mechanisms and factors influencing these neuroendocrine effects need to be further investigated.

#### Meningeal lymphatic vessels

Recent findings have shown the presence of a traditional functional lymphatic system in the meninges that allows the drainage of fluid, macromolecules and immune cells from the CNS into the deep cervical lymph nodes [46–48]. Anatomically, lymphatic vessels composed of lymphatic endothelial cells line up along the dural sinuses and leave the CNS from the base of the skull, and more importantly, these cells express traditional lymphatic vessel cell markers, such as Prox1 and CD31 [1, 49]. Under normal conditions, immune cells (T cells, B cells and dendritic cells) are present in the meningeal lymphatics, suggesting that in the steady state, the meningeal lymphatics are involved in the transport of immune cells from the CNS meninges and cerebrospinal fluid [46]. In disease states, the brain can regulate the peripheral immune system by introducing brain tissue in situ products and immune cells, such as brain-specific antigens, into the peripheral immune system through meningeal lymphatics [50]. It has been demonstrated in a mouse model of multiple sclerosis that suppression of the T-cell inflammatory response and attenuation of CNS damage can be achieved through ablation of meningeal lymphatics [51]. It has also been demonstrated in Alzheimer’s disease that meningeal lymphatics can promote amyloid-B clearance [52].

For brain-specific antigens, immunohistochemistry revealed that neuron-derived antigens, such as microtubule-associated protein 2 (MAP2) and myelin basic protein (MBP), were found in the deep cervical lymph nodes of acute stroke patients [53]. These antigens can be presented by antigen-presenting cells (APCs) to the T-cell compartment of the lymph node and induce an autoreactive T-cell response [53, 54]. In addition, noninfectious immune responses against injured tissues are initiated after CNS injury; these patterns, known as danger-associated molecular patterns (DAMPs) [3], are a group of molecular determinants derived from cellular debris, intracellular proteins/enzymes, or nuclear DNA/RNA that are released from the cell after CNS injury [55]. Proteins with a greater impact on the peripheral immune system include high-mobility group box 1 (HMGB1), S100 and ATP [56]. HMGB1 is a nuclear protein that binds to DNA and regulates gene transcription [57]; it can be produced by damaged neurons after injury and survives activation of the immune system by binding to RAGE on microglia/macrophages, linking nerve injury to microglia/macrophages [58, 59]. Like HMGB1, S100B binds to RAGE on microglia/macrophages and stimulates the upregulation of nitric oxide (NO), IL-1β, NF-κB, tumor necrosis factor (TNF)-a, several chemokines (CCL3, CCL5 and CXCL12) and chemokine receptors (CCR1 and CCR5) [60–62]. The source and role of ATP are relatively widespread, as ATP can be produced by damaged vascular cells and blood cells in addition to neuronal cells [63], and ATP has been demonstrated to lead to worsened outcomes in numerous models of disease (ischemic brain injury, spinal cord injury, and autoimmune diseases, among others) [56]. For example, ATP can act on purinergic receptors such as P2 × 7 or P2 × 4, which can activate microglia and astrocytes, in turn stimulating the activation of inflammatory cells and the release of a range of inflammatory substances, including inflammasome vesicles [64, 65].

#### Stroke-induced immune depression syndrome (SIDS)

An important phenomenon after stroke is the systemic immunosuppressive effect, which usually manifests as splenic atrophy accompanied by increased apoptosis or cellular dysfunction [66], along with the suppression of numerous inflammatory factors, including IL10, IL-1b, TNF-α, and IL-6. This state of immunosuppression has been referred to as SIDS [67]. Although this may be intended to attenuate the inflammatory response in the CNS, it has become an important cause of poststroke infections [68, 69]. The benefits of immunosuppression have been demonstrated in numerous studies; for example, the suppression of the immune response by splenectomy before middle cerebral artery occlusion (MCAO) or neonatal hypoxia-ischemia reduces cerebral infarction [70] and is accompanied by a significant reduction in the number of infiltrating neutrophils and activated microglia in the injured brain [71]. Poststroke splenic irradiation has also been shown to significantly reduce cerebral infarction in rats [72]. The mechanisms of injury-induced immunosuppression are largely unknown, and cutting-edge literature suggests that this suppressive effect is likely related in several ways, as described previously [73–75]. It has been shown that sympathetic nerves suppress the inflammatory response by inhibiting natural killer T-cell activity through the action of norepinephrine in the liver [76]. In addition, studies in the SAH model have shown that the hypothalamic‒pituitary‒adrenal axis after hemorrhage can lead to splenic contraction [77], which is a very important feature of immunosuppression [78]. Specifically, SNS-induced splenic crumpling may be achieved through the activation of a1 adrenergic receptors on smooth muscle cells [79]. Further studies have shown that cerebral hemorrhage leads to a significant reduction in splenic-derived leukocytes and lymphocytes in the peripheral blood [80], and the greater the hemorrhage is, the more severe the splenic constriction, the greater the risk of late infection, and the worse the prognosis [81, 82]. In addition to its immunosuppressive effects, CNS injury affects many systems and organs. For example, in the brain-gut axis, the neuroinflammatory response during stroke can lead to dysbiosis of the gut flora [83], reduced motility and increased permeability [84, 85]. In turn, changes in gut microbiology can affect the inflammatory response in the CNS, e.g., leading to altered T-cell homeostasis, thereby exacerbating the inflammatory response and poor prognosis [86]. Studies in the renal field have shown that hemorrhagic stroke-mediated production of inflammatory mediators is a central mechanism contributing to renal insufficiency [87], but concomitant activation of cholinergic anti-inflammatory pathways in splenocytes can achieve renal protection [88]. In addition, activation of the hypothalamic‒pituitary‒adrenal (HPA) axis, sympathetic and parasympathetic neuromodulation, and dysregulation of the intestinal microbiota after hemorrhagic stroke may also contribute to cardiac damage, such as heart attack, congestive heart failure, cardiac arrest, and atrial fibrillation [89–91].

### Pathway by which peripheral immune cells enter the central nervous system (Fig. 2)

**Fig. 2:**
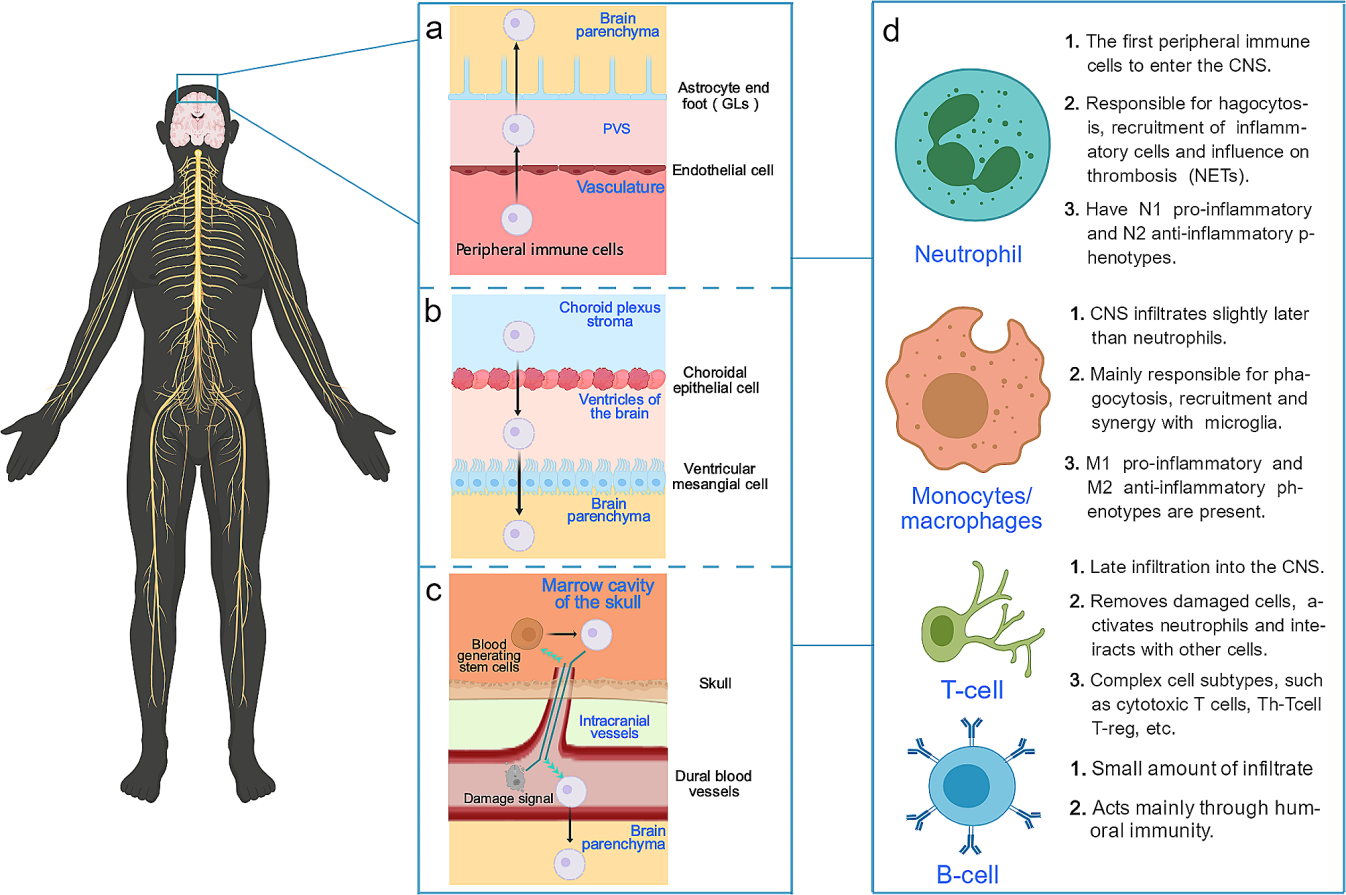
Pathways through which peripheral immune cells enter the central nervous system

#### Crossing the blood‒brain barrier

The most common route to cross the BBB is through small postcapillary veins, which has been confirmed by many studies. This process involves multiple steps: (1) After CNS injury, many peripheral immune cells are recruited to the cerebral vasculature due to the various effects described previously. (2) Depending on the action of P-, E-, and L-selectins, leukocytes initiate contact with the endothelium, reduce their velocity, and roll on the side of endothelial cells. (3) This reduction in velocity allows immune cells to pass through G protein-coupled receptors (GPCRs) and to recognize endothelial cell chemokines, which in turn activate adhesion molecules of the integrin family, resulting in the cell being firmly anchored to the luminal surface of the endothelial cell. (4) Finally, immune cells cross the BBB (which, strictly speaking, also requires the crossing of glial cell boundaries or glial limitans (GLs), the contingency barriers formed by astrocytes after injury) into the brain parenchyma through sites such as endothelial cell junctions [92–95].

Specifically, the BBB becomes permeable 3 min after injury [96], and even on the fourth day after CNS injury, the aggregation of neutrophils in the vasculature and the release of inflammatory factors are observed [97]; these changes are due to global vascular inflammation and are present in numerous models of stroke, including cerebral hemorrhage [98–100]. Vascular endothelial cells are rapidly activated after injury and upregulate numerous cytokines, including vascular cell adhesion molecule (VCAM-1), which promotes peripheral inflammatory cell adhesion and migration [101, 102]. Using molecular imaging techniques, researchers have found that this endothelial cell activation persists until at least 5 days postinjury in an ischemic infarction model, and this activation is global, as in acute ischemic cerebral infarction, as confirmed by positron emission computed tomography imaging of MMP9, which promotes BBB injury [103]. A similar effect has been demonstrated in the SAH model, where MMP9 released by neutrophils in the subarachnoid space damages the BBB [104], which leads to more peripheral immune cells infiltrating the brain parenchyma, which in turn leads to the production of more MMP9 and thus a vicious cycle [105, 106]. Numerous studies have demonstrated that attenuating the adhesion of neutrophils to endothelial cells via cell adhesion molecules (CAMs), such as VLA4 (a protein closely related to cell adhesion and migration), can significantly ameliorate the activation of microglia and neuronal damage in the brain parenchyma [97, 107]. In addition, IL-6 produced by neutrophils can exacerbate the disruption of tight junctions between endothelial cells in cerebral blood vessels, leading to an increase in BBB permeability and correlating with vasospasm [108, 109], suggesting that there is an outwardly activated immune mechanism from outside-in at the vascular/cerebral interface after SAH [110].

#### Core points of peripheral-central interactions in the choroid plexus

In addition to crossing the classic pathway of the BBB, there is a second important pathway through which peripheral immune cells enter the brain parenchyma. The choroidal plexus serves as a communication hub between the central nervous system and periphery, and under physiological conditions, there is a large circulation of immune cells in the cerebrospinal fluid (CSF), mainly in the choroid plexus, that generates CSF, which includes neutrophils, monocytes, T cells, B cells, and dendritic cells; this concept is supported by the recent discovery of meningeal lymphatic vessels in the CNS [111, 112]. This finding suggested that after primary CNS injury, the peripheral immune system contributes to secondary CNS injury by interacting with the CNS [113]. Indeed, after the onset of injury, immune cells cross the choroid plexus epithelium into the ventricles and then pass through the ventricular cell layer into the periventricular region [114]. The barrier of the choroid plexus epithelium is also often referred to as the BCSFB because of the tight network of connections that exist in the choroid plexus epithelium, thereby limiting the entry of cells and molecules from the stroma into the cerebrospinal fluid [115]. It has been demonstrated in experimental autoimmune cerebrospinal (EAE) inflammation models that early inflammatory cells originate from the choroidal stroma [116]. In addition, a marked increase in the number of macrophages, granulocytes and CD8^+^ T cells in the choroid plexus was found in multiple sclerosis patients [117, 118], and further studies have shown that this change is most likely due to the absence of the key tight junction protein claudin-3 [119]. In chronic diseases such as amyotrophic lateral sclerosis, Alzheimer’s disease and Parkinson’s disease [120], prolonged exposure to inflammatory factors such as reactive oxygen species (ROS) and dysregulated signaling between barrier cell types leads to increased BCSFB permeability, permitting the invasion of other peripheral immune cells, such as monocytes, into the CNS [121, 122]. Thus, the choroid plexus is a hub for peripheral-central nervous system interactions and plays an important role both in the surveillance and storage of inflammatory cells in the healthy state and in proinflammatory and anti-inflammatory effects after CNS injury.

#### Direct migration of skull bone marrow cells

Recent cutting-edge studies labeling local cells with spectrally resolved membrane dyes in models of ischemia and chemically induced acute brain inflammation have revealed the occurrence of a more rapid mode of neutrophil entry into the brain parenchyma: medullary-like cells (especially those of cranial bone marrow origin) migrate toward the inflamed brain through microscopic channels through the cranial endothelium, thereby directly connecting the cranial bone marrow cavity to the dura mater [123], and the relaxation of the extracellular matrix by proteases released by activated mast cells and intradural macrophages may also help in this process [124–126]. Anatomically, most of the blood vessels within the skull are located at the parietal bone, and the average diameter of these vessels is approximately 1.5 mm, which can be observed by corrosion casting of the human body. Corrosion casting shows that these blood vessels have many branches toward the bone marrow cavity [127, 128] and connecting to the meningeal vascular system [129], which is precisely the site of the early inflammatory immune response in many chronic diseases, such as Alzheimer’s disease [130]. Following CNS injury, these vessels can act as bidirectional conduits, and injury signals may preferentially reach the cranial bone marrow and directly cause local alterations in the proliferation and differentiation of hematopoietic stem cells, which further results in bone marrow-derived leukocytes being stronger than other sources of leukocytes in terms of both their inflammatory properties and their interactions with other cells [131–133]. There is also recent evidence that immune cells, such as neutrophils, can enter the subarachnoid space through head microvessels or meningeal vessels near the site of brain injury [134]; these cells in turn enter the brain parenchyma through GLs, although recent studies have been conducted mainly on T cells and disease models [135, 136]. Notably, recent studies have shown that cerebrospinal fluid can enter deep cervical lymph nodes not only from meningeal lymphatics but also from the nasopharyngeal lymphatic plexus through sieve lymphatics and that lymphatic endothelial cells show a variety of aberrant features with age, with exponential increases in the number of phosphorylated tau and apoptotic cells and a marked decrease in the amount of cerebrospinal fluid discharged. In conclusion, in-depth research is needed to determine how to target peripheral immune cells to support a more rapid, direct, and effective pathway to enter and thus influence the CNS.

### Effect of aging on brain–peripheral crosstalk

Stroke is largely a disease of aging. In fact, the incidence of stroke doubles every decade after age 55 or older, and 80% of strokes occur in people over 65 years of age [137, 138]. Moreover, stroke disease in the older age group (which often represents a greater biological age) also exhibits more severe damage, slower recovery, and higher mortality rates [139]. Studies have shown that aging leads to significant and complex changes in the immune system [140] and that low-grade chronic inflammation (inflammation) associated with aging increases the risk of stroke [141]. This alteration of the immune system plays a key role in secondary brain damage after stroke and leads to a poorer prognosis in older patients [142].

Aging has been shown to affect the interaction between the central nervous system and the peripheral immune system after stroke. Aging is a state of irreversible replication arrest and leads to changes in gene expression and phenotype [143]. Researchers have proposed an important concept called the “senescence-associated secretory phenotype”, whereby aging brain cells release more proinflammatory signals, such as IL, IL-1α, IL-1β, IL-6, and IL-8, which amplify the inflammatory response after stroke in elderly patients [144]. In addition, the intrinsic immune cells in the CNS are also affected by senescence, thereby affecting the immune microenvironment. For example, microglia are in dynamic equilibrium under normal conditions, where activation and inhibitory signals regulate each other, but with increasing age, the interaction of inhibitory signals, such as CD200, CXCL1, and CD47, with ligands is disrupted, which leads to the overactivation of microglia [145]. It has been shown that the expression level of transforming growth factor β (TGF-β) further increases with age, and TGF-β not only attenuates the level of anti-inflammatory cytokines secreted by microglia [146] but also inhibits the conversion of microglia from a proinflammatory phenotype to an anti-inflammatory phenotype by decreasing interferon-regulating factor 7 [147]. Further studies have confirmed that amyloid beta protein (Aβ) accumulates in the CNS during aging, which also leads to increased levels of proinflammatory factor secretion by microglia [148, 149]. In addition to directly affecting the inflammatory response in the CNS, aging also affects the way peripheral immune cells enter the CNS. The blood‒brain barrier, an important barrier protecting the brain parenchyma, contains vascular endothelial cells as its main constituent cells, and as endothelial cells are transformed to a senescent phenotype, endothelial cells are coupled to neurovascular contacts by attenuating the regulation of vascular endothelial growth factor (VEGF) and through the ROS/NO axis [150]. More importantly, endothelial cells of the senescent phenotype can also promote peripheral immune cell infiltration by upregulating proinflammatory factors (e.g., IL-6 and IL-1β) and vascular adhesion molecules (e.g., VCAM1), and this infiltration further impairs the barrier function of the BBB [151, 152]. In the blood cerebrospinal fluid barrier, tumor necrosis factor (INF) signaling in senescent choroidal cells impairs brain function, and at the same time, more monocyte-derived macrophages enter the brain parenchyma through the blood cerebrospinal fluid barrier [153]. Moreover, it has been demonstrated in aged mice that the meningeal lymphatic system also shows impairments such as reduced area coverage, smaller diameter and reduced cerebrospinal fluid drainage to the deep cervical lymph nodes with age, which may be associated with the downregulation of lymphangiogenic factor signaling in aging meningeal lymphatic vessel endothelial cells [52]. In conclusion, many studies have confirmed that stroke affects not only the signal secretion pattern of the central nervous system after stroke but also the way and pathway through which peripheral immune cells enter the central nervous system, and future studies on the effects of aging on the interaction between the central nervous system and the peripheral immune system after stroke are of great clinical significance.

## Infiltration of peripheral inflammatory cells and their crosstalk with the central nervous system

The CNS has long been viewed as an immune-privileged system, but this perceived paradigm has slowly changed over the past two decades [154], and it is clear from the previous section that the peripheral immune system plays a critically important role through its interaction with the CNS after CNS injury [155, 156]. A large body of evidence suggests that the immune system maintains a constant state of immune surveillance, looking for signals not only from external pathogens but also from damaged tissues, especially in the case of aseptic injuries such as traumatic brain injury (TBI), spinal cord injury, and stroke [1]. Indeed, the initial inflammatory response to CNS injury may be a mechanism of the innate immune response characterized by the initial production of DAMPs, the production of inflammatory cytokines/chemokines by resident innate immune cells (microglia and astrocytes) and the subsequent recruitment of infiltrating innate immune cells. These infiltrating innate immune cells include granulocytes (basophils, eosinophils, and neutrophils), monocytes (which subsequently differentiate into macrophages), T cells, B cells, and others [9]. Both CNS resident immune cells and infiltrating immune cells play a beneficial/harmful role in the immune response after injury, depending on multiple influences, such as the type of injury, the time of day, and even individual differences, such as age, which is corroborated by the effects observed in multiple sclerosis, Alzheimer’s disease, and other diseases through the blocking of immune or inflammatory responses [157, 158].

### Neutrophils

Neutrophils, one of the most widespread and common types of immune cells in the peripheral immune system, are the first cells to mobilize to the CNS after CNS injury, and they play important roles in the inflammatory response [159]. Neutrophils are the first to respond in the first few hours after injury, with a marked increase in lifespan and number that lasts up to 48 h [160, 161], and the infiltration of neutrophils can be observed as early as 30 min postinjury and peaks at 2–3 days [162]. However, there is also evidence that during this period (especially within 24 h), neutrophils in the human brain mainly accumulate in blood vessels and perivascular spaces and start to infiltrate into the brain parenchyma only 3–5 days after injury [163, 164]. This finding was further supported by a recent flow cytometry study in which only 40% of leukocytes in the brain parenchyma were derived from blood at 24 h after cerebral hemorrhage, and almost all of them were derived on day five [165]. Notably, the evidence that neutrophils accumulate in blood vessels rather than in parenchymal tissues after stroke is controversial, and more in-depth studies are needed at this time. Over time, neutrophils are gradually phagocytized by macrophages and microglia; thus, they gradually decrease in number [166]; however, the number of neutrophils in the brain parenchyma does not return to normal until the seventh day [167], and even up to 32 days after stroke, neutrophils can still be detected [168]. This late detection of neutrophils raises the possibility that neutrophils may be confined to the subarachnoid space due to changes in BBB reconstruction and cerebrospinal fluid flow, which leads to the degranulation of neutrophils and the release of inflammatory factors throughout the CNS, possibly leading to delayed brain injury [169].

The role of neutrophils in phagocytosis and inflammatory factor release has been extensively studied, and many factors, including interleukins (IL-6, IL-1α, IL-1β, and IL-8), TNF-α, LFA-1, leukotrienes, arachidonic acid, vWF, matrix metalloproteinase (MMP-9) and VEGF [170–172], have been identified as DAMPs associated with neutrophils, suggesting that neutrophils play numerous roles in the CNS. In addition, recent research has shown that neutrophil-released neutrophil extracellular traps (NETs) play important roles in both thrombosis and trapping invading microorganisms [173, 174]. In response to the stimulus of injury, a large meshwork of structures containing histones, DNA, and granule proteins, among others [175], is formed, and this meshwork may be a central crossroads of platelet aggregation, microthrombosis, and microglia-mediated inflammation [176, 177] and thus play an important role in irreversible impairment of the microcirculation [178].

Neutrophils also play an important role in interactions with other cellular components. In the TBI model, neutrophil depletion attenuates brain edema and microglial activation [179], and similar results have been found in CCL2 and CXCR2 gene deficiency models [180, 181]. On the other hand, microglia can be activated by neutrophils and blood catabolic products, increasing CXCL2 expression and interacting with CXCR2 on the surface of neutrophils [182], thereby recruiting more inflammatory cells into the subarachnoid space through chemoattraction and facilitating the clearance of blood substances, which is beneficial for neuronal recovery [169, 183, 184]. Moreover, although neutrophils act as phagocytes, the release of ROS (which are necessary for immune defense) via NADPH oxidase and myeloperoxidase [185] leads to the activation of microglia, the amplification of inflammatory processes, and a poor prognosis [186–188]; therefore, the elimination of free radicals in cerebral hemorrhage has demonstrated potential therapeutic benefits [189]. With the intensive study of microglia–neutrophil interactions, Neumann et al. provided the first in vivo evidence of physical interaction between microglia and infiltrating neutrophils for the first time in 2014, in which infiltrating neutrophils could induce microglial morphology alterations directly by physical contact [107, 190], thus enabling the capture of infiltrating neutrophils.

### Mononuclear/macrophages

The functions of monocytes/macrophages during injury include killing pathogens by phagocytosis, expressing inflammatory cytokines and enzymes, and subsequently producing ROS and reactive nitrogen species (RNS) [191]; monocytes/macrophages can also differentiate into inflammatory dendritic cells (DCs) or macrophages during inflammation [192, 193]. In rodents, two circulating monocyte subpopulations are known to exist in the peripheral immune system: “inflammatory monocytes” and “patrol” monocytes. “Inflammatory” monocytes are characterized by high levels of lymphocyte antigen 6 complex site C (LY6C), low to moderate levels of CX [3]-C motif chemokine receptor 1 (CX3CR1), and C-C chemokine receptor type 2 (CCR2) [194, 195]. These cells are actively recruited to the site of injury after CNS injury, where they promote an inflammatory response that increases significantly at 3 days postinjury [196, 197]. “Patrolling” monocytes, characterized by low levels of Ly6C, high levels of CXC3CR1, and no CCR2 [198, 199], have a relatively long lifespan, “patrol” the vascular lumen and contribute to the maintenance of vascular homeostasis, with peak numbers occurring at 6 days postinjury; additionally, these cells are actively recruited to the site of injury and contribute to the inflammatory response, with a significant increase occurring at 3 days postinjury. The number of invading cells peaks at 6 days after injury [200, 201].

Numerous studies have also confirmed the strong association between monocytes and other immune cells. Previous studies have demonstrated that CD16^+^ monocytes can lead to a threefold increase in APC activity to the original level in peripheral blood [202] and can promote T-cell proliferation [203]; specifically, monocytes can act as APCs along with dendritic cells, thereby activating T cells [204]. After TBI, myelin basic proteins, glial fibrillary acidic proteins, and other proteins produced as a result of brain cell injury are recognized by APCs and enter the lymphatic system through meningeal lymphatic vessels to activate T and B cells through antigen presentation [46].

In the CNS, microglial populations are renewed through local expansion and replenishment of circulating monocytes [205]. The activation of microglia after cerebral ischemia precedes and can be facilitated by blood-derived macrophages [206, 207], which are not recruited to the site of injury until 3–4 h after stroke [208], but their numbers remain low until the next day, with peak macrophage aggregation occurring on the seventh day after stroke [209, 210]. Conversely, monocyte/macrophage infiltration also further activates microglia, and Bhalala et al. demonstrated that it is possible to suppress peripheral immune cells by splenectomy, thereby attenuating the inflammatory response of microglia in the brain parenchyma [211]. In an in-depth study utilizing the MCAO mouse model, the differences in gene expression between blood-derived macrophages and microglia at 7 days poststroke were analyzed. Blood-derived macrophages preferred the M2 phenotype, and the expression of genes primarily involved in migration, proliferation, and calcium signaling was upregulated [212]. Using flow cytometry, Li distinguished between peripherally derived monocytes/macrophages and microglia and reported that NADPH-2, IL-1b and CD68 levels were greater in macrophages, whereas TGF-β1, IL-6 and TNF-a levels were greater in microglia [213]. It can be hypothesized that although microglia are rapidly activated and function in the acute phase, macrophages may be key factors exacerbating the inflammatory response. A recent study revealed the beneficial effects of the combination of macrophage colony-stimulating factor (M-CSF), IL-6, and transforming growth factor-beta (TGF-β), particularly in terms of cosecretion by microglia, macrophages, and endothelial cells [214].

Moreover, monocytes entering the CNS can be activated by DAMPs such as ATP, ADP and clone-stimulating factors, which results in activated monocytes/macrophages recruiting more neutrophils and monocytes to the site of injury via neurotrophic factors (e.g., BDNF, NGF) and chemotactic factors [215]. In turn, neutrophil depletion can reduce monocyte/macrophage infiltration [216]. Moreover, in a spinal cord injury model, peripheral macrophages can instead inhibit microglial activation [217], which once again demonstrates the dual-edged sword of characterizing inflammatory cells. However, the effects of the monocyte/macrophage subpopulation and age have yet to be thoroughly investigated [218]. For example, it has been demonstrated that, compared with adults, children under 10 years of age have less monocyte chemotaxis, which may reduce monocyte damage after traumatic brain injury [219, 220].

### T cells

Several recent studies have clearly demonstrated that T cells can act as patrolling lymphocytes, even in a healthy state, and that T cells can continuously monitor the brain and migrate toward the healthy brain, whereas subsequent interactions with brain-resident cells [135] are important for the maintenance of physiological function and plasticity [221–223]. Specifically, T cells have been shown to influence neural cell genesis [224]. T-cell entry into the subarachnoid space and perivascular space has been shown to be achieved by transendothelial cell migration through small postcapillary veins [95]. In the healthy state, however, patrolling T cells eventually terminate their patrols and enter deep cervical lymph nodes rather than the brain parenchyma due to a lack of antigen recognition [225]. This concept is supported by the activation of the immune response in the deep cervical lymph nodes by antigens derived from brain tissues [217, 226], although this immune response is more biased toward a B-cell-mediated humoral immune response, probably to inhibit the cytotoxicity of T cells [227, 228]. Through this mechanism, lymphocytes in the brain can be replaced in a healthy state in only approximately 12 h. Researchers have found that there are also many T cells, mainly CD4^+^ T cells, in the choroid plexus of the healthy brain [229]; these T cells may provide a cellular pool for T-cell responses to injury during brain injury. There is growing evidence of the important role of adaptive immunity, particularly T lymphocytes, in brain injury secondary to ischemia [230–232]. After cerebral hemorrhage, flow cytometry showed that CD4^+^ T-cell infiltration was significant and peaked on day 5 [233], whereas in cerebral ischemia, T-cell infiltration, especially that of CD8^+^ T cells, was lower than that in cerebral hemorrhage [234].

T cells are important for interacting with other immune cells. T cells may act on glial cells such as microglia and astrocytes to alter the brain microenvironment [235]. Different cytokines produced by different T-cell subsets may not have the same activating effect on microglia [236]. Upon activation, microglia can promote neural precursor cell (NPC) proliferation by secreting IGF-1 [237] and promoting oligodendrocyte formation [238]. Studies of T cells on astrocytes have shown that inflammatory factors produced by T cells can induce astrocytes to produce neutrophil chemokines, thereby promoting neutrophil infiltration [239]. In turn, the production of IL-15 by astrocytes and microglia after ischemic stroke has been shown to activate CD8^+^ T cells and NK cells and to accumulate in the brain parenchyma [240]. Findings from studies in humans support that IL-15 significantly enhances the cytotoxic effect of CD8^+^ T cells on target organs in the target tissues of patients with rheumatoid arthritis [241, 242], autoimmune myositis [243], obesity [244], celiac disease [245], and multiple sclerosis [246, 247], which is thought to be one of the mechanisms that leads to tissue destruction. Moreover, as another T-cell subtype, Treg cells have been shown to play a primarily protective role after injury. Specifically, in addition to directly suppressing the increase in inflammatory factor levels, Treg cells can also act directly on astrocytes and microglia to convert them to an anti-inflammatory phenotype [248]. In addition, Treg cells directly signal oligodendrocyte progenitors to differentiate into myelin-producing oligodendrocytes, and mature oligodendrocytes bind to demyelinated axons and rewrap these axons with myelin to restore nerve function and neuroprotection [249]. In addition to their ability to recruit cells [250], T cells can also cause microvascular dysfunction by binding to endothelial cells and platelets [251], which in turn impairs neuronal function. More in-depth studies have shown that the interaction of specific regulatory T cells with endothelial cells may be achieved through LFA-1/ICAM-1 signaling [252]. Therefore, it is more logical to put forth more effort into preventing thrombosis by blocking T cells and thus thrombosis than working on means of thrombolysis after thrombosis. Studies on T cells and monocytes/macrophages have shown a significant increase in ipsilateral thalamic CD4^+^ and CD8^+^ T cells by flow cytometry 14 days after stroke, and the sites of T-cell aggregation overlap with the areas of activated macrophage aggregation, which suggests that there may be interactions between T cells and macrophages [253]; however, there is also evidence that T-cell-produced IL-4 can promote the conversion of macrophages to the M2 phenotype, thereby suppressing inflammation [254, 255].

### B cells

There is a relative paucity of studies on the role of B cells after CNS injury, which may be related to their low infiltration rate after injury [165]. Up to 8 weeks after MCAO in mice, B cells migrate to distal brain regions (e.g., the dentate gyrus, hypothalamus, olfactory regions, and cerebellum), which is associated with neurogenesis and functional recovery and has specific positive effects on motor recovery [256]. This migration may explain and support the results of another study in which motor recovery improved despite continued thalamic and hippocampal degeneration and associated cognitive dysfunction [257], which has important implications for neurological recovery. Although the infiltration rate is low, the role of humoral immunity induced by B cells cannot be ignored [217], and B cells may be involved in brain injury by producing autoantibodies against antigens such as MBP and GFAP [217, 258]. For example, in a study of children (7–16 years old) with chronic posttraumatic headache secondary to mild craniocerebral injury, B cells were shown to produce autoantibodies against AMPA and NMDA receptor subunits up to one year after injury, thus leading to a poor prognosis [259]. Although B cells play a minimal role in the formation of focal brain injury in the acute phase [260, 261], analysis of CSF composition has confirmed that B cells can mediate the activation of humoral immunity during stroke [217] and cocluster with the immunoglobulins they produce in the thalamus, which in turn leads to cognitive dysfunction [262].

## Peripheral inflammatory cells participate in the pathophysiological process after stroke

### Ischemic stroke

In ischemic stroke, the primary injury is the sudden cessation of blood supply to the vascular region, leading to neuronal cell death and the formation of an ischemic core surrounded by a hypoperfused area known as the hemidiaphragm, and peripheral immune cells play an important role in the secondary injury caused by the primary injury [263, 264]. Studies have confirmed that neutrophils are the predominant cells in the ischemic hemisphere 3 days after MCAO [265]. Increased neutrophils are positively correlated with infarct volume and functional defects [162]. More importantly, they can contribute to tissue damage by releasing elastase, which destroys thin-walled tissue, and by producing reactive oxygen species that lead to BBB destruction [266]. The elevation of neutrophils may be due to increased release from the bone marrow and spleen along with decreased apoptosis of neutrophils [267]. In contrast to patients with neutrophils, patients with ischemic stroke have a reduced number of lymphocytes and therefore an elevated neutrophil ratio. This ratio is strongly correlated with infarct size and mortality [268]. However, despite their association with tissue damage, there is no clear correlation between the number of circulating neutrophils and the extent of ischemic injury [269], suggesting a possible beneficial effect of neutrophils. This idea was first proposed in the field of oncology research, where by analogy to the M1M2 phenotype of macrophages, researchers found that neutrophils also exist in different proinflammatory or anti-inflammatory subtypes, where the proinflammatory type was named N1 and the anti-inflammatory type was named N2 [270] and that TRL4 of the TLR family promotes the conversion of neutrophils to the N1 phenotype [271], while rosiglitazone promotes the conversion of neutrophil granulocytes to the N2 phenotype [272]. Furthermore, in the MCAO model, investigators found that type N2 neutrophils are more easily and efficiently cleared by microglia than other subtypes, which provides new ideas for future treatment [273, 274].

Monocytes also play an important role in cerebral ischemia. Sensory-motor functional CCR + monocytes/macrophages are not only associated with the maintenance of neurovascular unit integrity but also help to induce the migration of neuroblasts from areas of neurogenesis to the site of injury [275]. This may explain why CCR + monocytes can promote the recovery of sensorimotor nerve function in the first week after cerebral ischemia [276]. In the MCAO model, the phenotypic transformation of CCR2 + monocytes to a more activated state after infiltration into ischemic tissues was found at 14 days postinjury, suggesting that monocytes/macrophages play a pleiotropic role in mediating the injury and repair process after cerebral ischemia [277]. Because blood-derived monocytes/macrophages are morphologically and functionally very similar to resident microglia in the brain parenchyma, finding specific labeling methods to differentiate between the two cell types is a difficult challenge [278]. The excellent work of Tanaka et al. using chimeric mice with enhanced green fluorescent protein (GFP) bone marrow provides a useful tool to distinguish between resident macrophages and blood-derived macrophages in ischemic brain injury [206], and further studies have shown that it is also possible to explore differences in CD45 expression between microglia and monocytes/macrophages using flow cytometry to differentiate [279, 280]. These studies once again confirmed that blood-derived macrophages infiltrate later than microglia, but this, on the other hand, suggested a possible role for blood-derived macrophages in tissue remodeling [281]. This approach has important clinical and therapeutic applications because circulating monocytes are more receptive to drug manipulation than microglia located behind the BBB.

T cells, in contrast to neutrophils and monocytes/macrophages, have rarely been reported to be present in the vasculature or brain parenchyma within 24 h of injury and are recruited into the brain parenchyma mainly in the later stages of injury [261]. Experimental results in experimental cerebral ischemic injury models (including the MCAO model and the photochemically induced focal ischemia model) have shown that T-cell infiltration does not appear in large numbers until the third day and is mainly concentrated in the marginal zone of the lesion and that the number of T cells gradually increases between the third and the seventh day [282, 283]; T-cell infiltration is still detected in the brain parenchyma even for at least 30 days after cerebral infarction [284]. It has been shown that T-cell deletion has a neuroprotective effect in a focal cerebral ischemia model and that this effect disappears after T cells are retransplanted into immunodeficient mice [285, 286]. Many intensive studies have investigated different T-cell types, including CD4 + T cells, CD8 + T cells and Tregs [287], and especially in the delayed brain injury phase, an increasing number of studies have attempted to intervene in Treg cells because Treg cell infiltration occurs significantly later than that of other T-cell subsets and because of the neuroprotective function of Tregs [288]. During neuroregeneration from ischemic injury, the number of Tregs in the lateral ventricle of the ischemic hemisphere is increased and correlates with the proliferation of NPCs [289], which suggests that T cells may play a role in the migration of NPCs to the site of injury to repair damage [290, 291]. This is corroborated by the fact that in the healthy state, T cells can come into direct contact with NPCs (neural precursor cells) and interact with them functionally [292]. It has been shown that the removal of Tregs using CD25 on the seventh day after MCAO-induced cerebral ischemia in mice leads to increased neuronal damage [293, 294], and clinical studies have confirmed that increasing the number or function of Tregs can have a neuroprotective effect [287]. In terms of neuroprotective effects, researchers have demonstrated in stroke models that the mTOR inhibitor rapamycin protects neurons through the anti-inflammatory effects of Tregs [295, 296]. However, different methods for inhibiting Tregs have led to controversial results [297].

### Hemorrhagic stroke

Hemorrhagic stroke can include two major types of cerebral hemorrhage and subarachnoid space hemorrhage, in which the primary brain damage caused by cerebral hemorrhage is caused mainly by the rupture of blood vessels, leading to the extravasation of blood components directly into the brain and the formation of hematomas, which cause structural damage. However, whether cerebral ischemia occurs after hemorrhage is controversial, as is whether this change is correlated with the severity of hemorrhage, etc [9]. The most immediate damage after SAH consists of two main events: (1) sudden blood invasion in the subarachnoid space and (2) hypoxia due to cerebral circulatory disturbances and increased intracranial pressure [178]. However, a growing number of studies have confirmed the importance of secondary injury caused by inflammatory infiltration in terms of mortality and neurological prognosis. The development of secondary brain injury involves numerous factors, such as the induction of neuroinflammatory factors produced by hemolytic products [298] and the effects of metabolites produced in situ by brain tissue [299, 300], all of which are inextricably linked to inflammatory cell infiltration [301].

Vasospasm is considered one of the strongest risk factors for secondary brain injury after hemorrhagic stroke and is significantly associated with delayed cerebral ischemia (DCI) [172]. In patients with vasospasm, the number of neutrophils and their enzymes myeloperoxidase and NADPH oxidase are greater [170, 302], and vasospasm is significantly improved if neutrophils are depleted or the CD11b/CD18 complex is blocked [303]. In addition, neutrophil surface Toll-like receptor 4 (TLR-4), TLR-related interferon activator (TRIF) and myeloid differentiation primary response gene (MyD88) are important mediators of neuronal apoptosis and cerebral vasospasm, respectively [304]. Although 70% of angiograms after aneurysmal SAH show vasospasm, the incidence of DCI is in fact only 30% [305], and more importantly, even when the incidence of vasospasm is reduced by pharmacological treatment, clinical outcomes are not improved [306]. This finding suggested that factors such as neuroinflammation, BBB disruption, and microthrombosis should be considered in addition to vasospasm [307] and may lead to secondary delayed cerebral ischemia or secondary brain injury [308, 309]. As a means of detection, neutrophil counts are correlated with the occurrence and prognosis of DCI, both in the early stages of injury [310] and three days after the onset of aneurysmal SAH [311]. In addition to these findings [312], there is evidence that NLRs are correlated with neurological symptoms such as amnesia and the presence of CT positivity in lesions [313]. However, due to the complexity of the etiology and progression of DCI, there is still a long way to go in predicting or diagnosing DCI or delayed brain injury by using inflammation as an entry point, and as yet, there is no method that can be repeated many times [172]. Another important factor in DCI is thrombosis, and neutrophils are incredibly perceptive of thrombi; even in reverse blood flow, the movement of neutrophils can be achieved in vessels without thrombi, which may explain why the number of neutrophils is ten times greater than that of other lymphocytes [231]. This strong ability to cope with shear stress may be achieved by sticky membrane slings [314]; unfortunately, the resolution of in vivo imaging is not sufficient to visualize these structures. Future studies could focus on identifying inflammatory cells with microthrombi, delayed axonal degeneration, etc [315].

Within the secondary injury phase after hemorrhagic stroke, although many of the physiological changes in the acute phase, such as cerebral hemorrhage and increased intracranial pressure, gradually subside and the subarachnoid space is progressively cleared of clots, a second focus of neuronal damage occurs between 14 and 28 days after hemorrhage [316]. This second focus may be related to a second vasospasm accompanied by thickening of the vascular wall that occurs at the end of the acute phase [303, 317]; however, ischemia alone is not sufficient to cause neutrophils to cross the BBB [318]. Although the current literature includes a series of studies on the mechanisms that may lead to delayed neurological deficits (DNDs) after hemorrhagic stroke, the main focus has been on cytokines [308], including the inflammatory cytokine profile in the brain and periphery and their impact on vascular dysregulation [110], and on the role of inflammatory cells in DNDs revolving around the association with vasospasm only; however, there is a lack of relevant evidence for the direct role of inflammatory cells in DNDs [319]. Studies have demonstrated that neutrophils play a key role in the progression of DNDs; that neutrophils modulate the expression of NMDAR subunits in the hippocampus, thereby leading to spatial memory deficits; and that neutrophil depletion can eliminate DNDs [320].

BBB disruption after hemorrhagic stroke leads to the infiltration of inflammatory cells [321, 322], among which monocytes play an important role and are the second peripheral immune cells to enter the CNS after neutrophils [161]. After neutrophil invasion, monocytes adhere to the vessel wall and move toward the ischemic area. Blood-derived monocyte/macrophage counts reach a peak at 1–2 days after injury [323], reach maximal activity at 3–7 days [162] and persist for several weeks [324]. Blood degradation products after hemorrhagic stroke are sufficient to activate [184] the release of ET-1 [325, 326]. In addition, monocyte infiltration has been associated with elevated levels of many inflammatory factors, such as CXCL-1, CXCL-9 and CXCL-10, which have been shown to be associated with DCI after aneurysmal SAH [327]. However, a change in cytokine concentration is positively correlated with a change in blood flow velocity [328]. This process results in a vicious cycle in which monocytes produce cytokines, leading to vasoconstriction; blood flow slows, and cytokine concentrations rise, leading to further vasoconstriction. Monocytes infiltrate the brain parenchyma after CNS injury, activate macrophages and initiate the phagocytosis and degradation of cellular products [184]. The current view is that there are two phenotypes of macrophages, M1 proinflammatory and M2 inhibitory inflammatory types, and that the M1 type is associated with increased production of proinflammatory cytokines (e.g., TNF-α, IL-1b, and NADPH), which ultimately leads to inflammation in the nervous system [329, 330]. The macrophage phenotype is regulated by a variety of factors; for example, blood metabolites such as hemoglobin and iron promote macrophage conversion to the M1 type [331]; a variety of factors such as metabolite clearance promote macrophage conversion to the M2 type [332, 333]; and numerous efforts have been made to promote monocyte/macrophage phenotypic conversion to the M2 type. For example, studies have shown that atorvastatin and cordycepin inhibit microglial/macrophage proinflammatory polarization and promote anti-inflammatory polarization after TBI. This effect may further inhibit BBB disruption and neutrophil infiltration [334, 335].

After hemorrhagic stroke, activated T cells infiltrate the brain parenchyma and contribute to neuronal damage and poor prognosis by releasing ROS and cytokines [336]. In addition, cell-based CD28 expression in the adaptive immune system is a coactivation signal for T cells, leading not only to increased CD4 + and CD8 + T-cell activation [337] but also to increased cytokine production by T cells [338]. T-cell accumulation can be observed in the thalamus distal to the primary injury site, which has been shown to undergo secondary neuronal degeneration, and it has been found that the infiltration of T cells continues at least two weeks after stroke, with more CD4 + T cells than CD8 + T cells [253], which coincides with the severe period of poststroke secondary neurodegeneration (SND) [339]. In the middle to late stages of DCI, CD3 + Tregs are significantly elevated compared to those in the EBI stage and are significantly associated with infection after SAH [340]. This may be because Tregs inhibit T-cell proliferation and reduce the secretion of cytokines (e.g., IL-10 and TNF-α), thereby attenuating the inflammatory response [341, 342]. In addition, Tregs also protect against BBB damage, which is mainly achieved by inhibiting peripheral MMP-9 production [343]. These findings show that the role of T cells in the development of inflammation is bidirectional, as cytokines can both eliminate damaged cells and clear microorganisms, leading to a severe inflammatory cascade [344, 345]. Angiogenesis is an important repair mechanism after stroke, and the effect of T cells on angiogenesis has been demonstrated in numerous organs. In tumors and lung ischemia, proinflammatory Th1 cells are thought to inhibit vascular growth, whereas anti-inflammatory Treg cells are associated with increased angiogenesis [346]; moreover, these various facilitating effects are reflected not only by the fact that Treg cells can inhibit effector T cells but also by their active secretion of chemokines, vascular endothelial growth factor, and transforming growth factor to promote neoangiogenesis [347, 348]. In rat corneal tissue, T-cell-derived IL-17 has also been shown to promote neoangiogenesis [349]. Although the role of T cells in promoting angiogenesis has not been studied in stroke, this is not a valuable research direction.

## Potential treatment strategies and status evaluation by targeting brain–peripheral immune responses after stroke

The important role of inflammatory injury in CNS disease is receiving increasing attention, and treatments targeting inflammation have numerous advantages over other approaches (Table 1). First, anti-inflammatory treatments have an advantage in terms of time window limitations; in ischemic stroke, the window for thrombolysis is only 4.5 [350], but treatments targeting inflammation are effective 12–24 h after stroke [351]. In addition, inflammatory treatments are beneficial even for hemorrhagic strokes, as they are more broadly available and less demanding in terms of diagnosing the type of injury [352]. Finally, regardless of the type of CNS injury, the beneficial role of treatments targeting inflammation in ischemia‒reperfusion can help smooth the recovery period in patients treated for primary injury [353].

**Table 1:** Potential treatments targeting peripheral immune responses for central nervous system injuries

Drug	Drug target/type	Disease	Effector cell	Primary role	references
Fingolimod	Sphingosine 1-phosphate receptor	Ischemic Stroke	Microglia	Regulation of microglia M2 polarization Neuroprotective function Reduces volume of brain tissue damage	Proc Natl Acad Sci USA. 2014 Dec 23;111(51):18315-20. Circulation. 2015 Sep 22;132(12):1104-1112.
		SAH	Neutrophil	Significantly reduced intravascular leukocyte adhesion Improved neurologic prognosis after SAH in rats	J Neuroinflammation. 2015 Jan 27:12:16.
		ICH	T-cell NK cell	Blocking T or NK cell infiltration into the CNS Improves neurologic prognosis after SAH in rats	Neurosci Bull. 2015 Dec;31(6):755 − 62.
Minocycline	Antibiotics	Ischemic Stroke	Microglia	Resulted in a significant reduction in the patient’s MRS score	Neurology. 2007 Oct 2;69(14):1404-10. Stroke. 2010 Oct;41(10):2283-7.
		TBI		Inhibits microglia polarization to M1 type and promotes mechanization to M2 type Reduces the size of the injury site	J Neurotrauma. 2010 May;27(5):911 − 21.
		ICH	Microglia/macrophage	Decreased number of microglia and macrophages around the hematoma Decreased brain water content Reduced levels of TNF-α and MMP12	Acta Neurochir Suppl. 2010:106:147 − 50. Exp Neurol. 2007 Oct;207(2):227 − 37.
BI1002494	Splenic tyrosine kinase(SYK) inhibitor	Ischemic Stroke	Platelet	Inhibits thrombosis and reduces damage	Arterioscler Thromb Vasc Biol. 2016 Jun;36(6):1247-53.
Recombinant T-cell receptor ligand(RTL)	Recombinant T-cell receptor		T-cell	Reduced infarct size by 50% Reduced the number of immune cells recruited in the brain	Transl Stroke Res. 2016 Feb;7(1):70 − 8. Neuroscience. 2015 Mar 12:288:112-9.
Bexarotene	Retinoid X receptor(RXR)		N2 neutrophil	Increases the number of N2 neutrophils Neuroprotective effect	Pharmacol Res. 2015 Dec:102:298–307.
Bone marrow mononuclear cell(BMMCs)	/		Monocyte	Improved clinical functional prognosis	Cell Transplant. 2014:23 Suppl 1:S57-64. Stroke. 2016 Jul;47(7):1817-24.
Granulocyte colony-stimulating factor(G-CSF)	Granulocyte colony-stimulating factor receptor(G-CSFR)		Monocyte Microglia	Promotes microglia polarization to M2 type Promotes axonal germination	Stroke. 2012 Feb;43(2):405 − 11. Exp Neurol. 2015 Jan:263:17–27.
Recombinant human IL-1 receptor antagonists	IL-1 receptor		Multi-cell type	NIH Stroke Scale Scores and Modified Rankin Scale (mRS) Scores Increased	J Neurol Neurosurg Psychiatry. 2005 Oct;76(10):1366-72.
Natalizumab	α4-integrin		T-cell	Reduction of cerebral infarct volume	Brain. 2011 Mar;134(Pt 3):704 − 20. Stroke. 2014 Jun;45(6):1610-1.
Dexamethasone/hydrocortisone	Ras homolog family member (Rho)-A and-B proteins	TBI	Multi-cell type	Reduces the intensity of the immune response Reduces the number of circulating lymphocytes	J Neurotrauma. 2014 Apr 15;31(8):699–712. J Neuroinflammation. 2016 Aug 25;13(1):197.
Atorvastatin	HMG-CoA reductase		T-reg cell Microglia Macrophage	Neuroprotective effects Increases the number of Tregs Increases expression of IL-10	Neurocrit Care. 2017 Feb;26(1):122–132. J Neurotrauma. 2016 Aug 15;33(16):1492 − 500. J Neuroinflammation. 2017 Aug 23;14(1):167.
Anti-HMGB1 monoclonal antibody	HMGB1		Endothelial cell Microglia	Reducing disruption of the blood-brain barrier Reduces cerebral edema Limit inflammatory cascade	Ann Neurol. 2012 Sep;72(3):373 − 84. Stroke. 2011 May;42(5):1420-8.
Methylene blue	GABAA		Microglia	Improves blood-brain barrier integrity Reduces volume of damage Protects against neuronal death	Front Neurol. 2019 Nov 8:10:1133. Neuropharmacology. 2017 Jun:119:100–110.
Metformin	ERK1/2 p38-MAPK		Microglia Neutrophil Monocyte	Reduced microglia activation Decreased neutrophil/lymphocyte ratio	J Neurol. 2019 Aug;266(8):1988–1997. Brain Res Bull. 2018 Jun:140:154–161.
T-0080	Formyl peptide receptor 1(FPR1)	ICH	Microglia Neutrophil	Attenuates activation of neutrophils by microglia May reduce cerebral edema Improves neurologic prognosis after ich	Curr Neuropharmacol. 2021;19(9):1590–1605.
Rapamycin	mTOR		T-reg cell	Increase Tregs cells in brain tissue Improves neurological function after ICH	J Cereb Blood Flow Metab. 2017 Mar;37(3):967–979. J Neuroinflammation. 2014 Mar 6:11:44.
Celecoxib	Cyclooxygenase2		Multi-cell type	Reduction of inflammatory cell infiltration, cerebral edema and subsequent perihematoma cell death	J Cereb Blood Flow Metab. 2004 Aug;24(8):926 − 33. Eur J Neurol. 2013 Aug;20(8):1161-9.
Rosiglitazone	PPAR-γ/CD36		Microglia/macrophage	Facilitate hematoma clearance after ICH	J Neuropathol Exp Neurol. 2000 Aug;59(8):641 − 51. Int J Stroke. 2013 Jul;8(5):388 − 96.
IL-2	IL-2 receptor	SAH	Neutrophil	Suppression of brain pro-inflammatory factors and peripheral neutrophils Reduces neuronal damage	ACS Chem Neurosci. 2021 Feb 3;12(3):430–440.

### Ischemic stroke

There has been some progress in research targeting peripheral inflammatory infiltrating cells. First, a series of attempts have been made to treat ischemic stroke. In acute ischemic stroke, neurological recovery can be improved, and the volume of brain tissue damage can be reduced by oral administration of fingolimod [354, 355], which is attributed to the fact that fingolimod can promote the polarization of microglia to the M2 type [356, 357]. Another clinical study revealed that ischemic stroke patients could achieve better MRS scores at 90 days after taking minocycline orally and that minocycline is safe when combined with tissue plasminogen activator [358]. Splenic tyrosine kinase (SYK) is a nonreceptor tyrosine kinase found in organs such as the spleen and thymus and is closely associated with inflammation. SYK inhibitors protect against thrombosis and attenuate cerebral infarction injury [359], and more importantly, this effect does not affect the hemostatic effect of platelets. Recombinant T-cell receptors (RTLs) are a type of major histocompatibility complex (MCH) II that are predominantly present in the MCAO model in association with mouse brain tissue and the spleen. Treatment with its ligand recombinant T-cell receptor ligand (RTL) resulted in a 50% reduction in the infarct area and a reduction in the number of immune cells recruited to the brain [360, 361]. Moreover, researchers found that bexarotene, a retinoid X receptor (RXR) agonist, increased the number of N2 neutrophils in MCAO mice, which alleviated inflammatory damage and produced neuroprotection [362]. Bone marrow mononuclear cells (BMNCs), which include hematopoietic spectrum cells, stem cells, and progenitor cells, have been shown to promote neural stem cell proliferation [363, 364]; therefore, investigators explored the role of BMNC transplantation in ischemic stroke and confirmed that BMNCs are associated with improved neurological function [365, 366]. The two main types of grafts are arterial and venous grafts, and researchers have found that although arterial grafts are relatively risky, the grafts increase growth factor levels and decrease inflammatory factor levels. In contrast, vein grafts are safe but have no beneficial effects [367, 368]. Granulocyte colony-stimulating factor (G-CSF) can stimulate the infiltration of BMNCs to the site of injury after ischemic stroke [369], in addition to promoting axonal growth and the polarization of microglia toward the M2 phenotype [370, 371]; however, more studies are needed to confirm the exact efficacy of G-CSF in clinical translation. In a phase IIa clinical study of acute-phase ischemic stroke, investigators demonstrated by recombinant human IL-1 receptor antagonists that IL-1 receptor antagonists not only significantly improved patients’ NIH stroke scores three months after injury but also led to better performance on the modified Rankin scale (mRS) score [372]. In a study of natalizumab, investigators found that in acute ischemic stroke, natalizumab prevents T cells from entering the CNS by blocking α4-integrin, which is beneficial for reducing the volume of cerebral infarction [373].

### Hemorrhagic stroke

In hemorrhagic stroke, numerous strategies have also been adopted for the infiltration of peripheral immune cells. Clinical studies have shown that fingolimod not only works in treating ischemic stroke, as mentioned earlier but also has a therapeutic effect on hemorrhagic stroke. Fingolimod blocks the release of lymphocytes from lymph nodes, which results in fewer T cells and NK cells infiltrating the CNS [374]; moreover, fingolimod attenuates neutrophil adherence to blood vessels after SAH, thereby improving neurological function after SAH [375]. Minocycline also plays a therapeutic role in intracerebral hemorrhage (ICH), and studies have shown that on the fifth day after ICH, minocycline reduces the number of microglia and macrophages around the hematoma, attenuates cerebral edema and decreases the expression levels of TNFα and MMP12 [376]. Neutrophil recruitment to and activation in the CNS are associated with formyl peptide receptor 1 (FPR1) expression on microglia and are in fact mediated through IL-1β. The activation of neutrophils by microglia is attenuated by the FPR1 inhibitor T-0080, which reduces cerebral edema and improves neurological function after ICH [377, 378]. Similarly, myeloperoxidase (MPO) can mediate the activation of microglia against neutrophils, and MPO knockout can play a beneficial role in the recovery of neurological function after SAH; however, further investigation is needed to determine how these findings can be translated into clinical application [379]. The beneficial role of Treg cells in hemorrhagic stroke has been previously described. During the recovery phase of hemorrhagic stroke, rapamycin inhibits mTOR activation and increases the number of Treg cells in brain tissue; more importantly, these effects lead to increased levels of IL-10 and transforming growth factor-b, which are important for improving neurological function after ICH [380]. Moreover, multicenter clinical trial findings have confirmed that selective inhibition of cyclooxygenase 2 (COX2) using celecoxib after cerebral hemorrhage reduces inflammatory cell infiltration, cerebral edema, and perihematomal cell death [381]. Rosiglitazone has been shown to have a therapeutic effect not only on diabetes mellitus but also on ICH, after which it may act as an agonist of PPAR-γ and upregulate the expression of CD36, which has been shown to regulate the phagocytosis of microglia and phagocytes in ICH, suggesting that rosiglitazone can promote hematoma clearance after ICH [382]. In addition, it has been experimentally confirmed that low doses of IL-2 can attenuate neuronal damage after SAH, which is achieved by inhibiting proinflammatory factors and peripheral neutrophils [340].

## Perspectives and conclusion

Over the past two decades, there has been increasing interest in the interactions between the CNS and the peripheral immune system after CNS injury. Here, we summarize the effects of CNS injury on the peripheral immune system. CNS injury is signaled to the peripheral immune system through the autonomic nervous system, neuroendocrine system, and meningeal lymphatic vascular system, the activation of which allows peripheral immune cells to enter the CNS through the ruptured BBB and BCSFB and direct infiltration into the bone marrow of the skull to play a role in various acute CNS injuries. We conclude by summarizing the monitoring methods, clinical findings, and corresponding therapeutic advances for this interaction. A more complete description of the effects on the peripheral immune system after acute CNS injury and the effects of the corresponding inflammatory cell infiltration in secondary injury after primary injury is presented. Regarding the existing studies and future research priorities, we make the following points: (1) The inflammatory response is a complex network with a complex interplay of effects, both in terms of the relationship between the CNS and the peripheral immune system and in terms of the relationships between the different inflammatory components. Future studies of a particular inflammatory factor should not only be more in-depth but also explore the overall effects at the level of the network of inflammatory relationships. (2) The role of inflammatory cells in CNS injury is often a double-edged sword, and it is clear that simply promoting or inhibiting a factor in response to a certain factor does not have an absolute positive effect; for example, although the immunosuppressive effect or artificial immunosuppression after injury reduces the inflammatory response at the site of injury to a certain extent, it also leads to more infections, and ultimately, there is no clear evidence of a beneficial effect on prognosis or survival. Therefore, a combination of multifactorial therapies or interventions targeting beneficial effects should be considered for the treatment of inflammatory injuries in the future. (3) Although the inflammatory patterns induced by different CNS injuries are very similar, there are many factors affecting the inflammatory response, including not only different types of injuries but also different periods of disease, severity of disease, and even age; therefore, future research should be conducted to investigate the inflammatory response and treatment modalities. Therefore, the generalizability of the inflammatory response and treatment modalities should be further investigated in the future.

## Data Availability

No datasets were generated or analysed during the current study.

## Abbreviations

CNS: Central nervous system
BBB: Blood‒brain barrier
BCSFB: Blood–cerebrospinal fluid barrier
SAH: Subarachnoid hemorrhage
ANS: Autonomic nervous system
SNS: Sympathetic nervous system
PSNS: Parasympathetic nervous system
LPS: Lipopolysaccharide
AVP: Arginine vasopressin
ACTH: Adrenocorticotropic hormone
TSH: Thyroid stimulating hormone
FSH: Follicle-stimulating hormone
LH: Luteinizing hormone
GH: Growth hormone
MAP2: Microtubule-associated protein 2
MBP: Myelin basic protein
APCs: Antigen-presenting cells
DAMPs: Danger-associated molecular patterns
HMGB1: High-mobility group box 1
NO: Nitric oxide
TNF: Tumor necrosis factor
SIDS: Stroke-induced immune depression syndrome
MCAO: Middle cerebral artery occlusion
HPA: Hypothalamic‒pituitary‒adrenal
GPCRs: G protein-coupled receptors
GLs: Glial limitans
VCAM-1: Vascular cell adhesion molecule
CAMs: Cell adhesion molecules
VLA4: Very late antigen-4
CSF: Cerebrospinal fluid
EAE: Experimental autoimmune cerebrospinal
ROS: Reactive oxygen species
TBI: Traumatic brain injury
MMP-9: Matrix metalloproteinase
NETs: Neutrophil extracellular traps
LY6C: Lymphocyte antigen 6 complex site C
CX3CR1: CX(3)-C motif chemokine receptor 1
CCR2: C-C chemokine receptor type 2
M-CSF: Macrophage colony-stimulating factor
TGF-β: Transforming growth factor-beta
NPC: Neural precursor cell
DCI: Delayed cerebral ischemia
TLR-4: Toll-like receptor 4
TRIF: TLR-related interferon activator
MyD88: Myeloid differentiation primary response gene
DNDs: Delayed neurological deficits
SND: Poststroke secondary neurodegeneration
ICH: Intracerebral hemorrhage
FPR1: Formyl peptide receptor 1
MPO: Myeloperoxidase
COX2: Cyclooxygenase 2
